# Clinical, psychological, and hematological factors predicting sleep bruxism in patients with temporomandibular disorders

**DOI:** 10.1038/s41598-025-03339-3

**Published:** 2025-05-31

**Authors:** Yeon-Hee Lee, Suk Chon, Q-Schick Auh, Merel Charlotte Verhoeff, Frank Lobbezoo

**Affiliations:** 1 Department of Orofacial Pain and Oral Medicine, College of Dentistry, Kyung Hee University Dental Hospital, Kyung Hee University, #613 Hoegi-dong, Dongdaemun-gu, Seoul, 02447 Korea; 2 Department of Endocrinology, Kyung Hee University, Kyung Hee University Medical Center, #613 Hoegi-dong, Dongdaemun-gu, Seoul, 02447 Korea; 3 Department of Orofacial Pain and Dysfunction, Academic Centre for Dentistry Amsterdam (ACTA), University of Amsterdam and Vrije Universiteit Amsterdam, Amsterdam, The Netherlands; 4 Center for Systems Biology, Massachusetts General Hospital, Harvard Medical School, 185 Cambridge Street, Boston, MA 02114 USA

**Keywords:** Temporomandibular disorder, Sleep, Bruxism, Pittsburgh sleep quality index, Cortisol, Adrenocorticotropic hormone, Biomarkers, Diseases, Endocrinology, Medical research, Risk factors, Signs and symptoms

## Abstract

This cross-sectional observational study aimed to identify the predictors of sleep bruxism (SB) in patients with temporomandibular disorder (TMD) and to comprehensively investigate its association with clinical, sleep-related, psychological, and hematological factors. Seventy-nine patients with TMD (69 females and 10 males; mean age 45.46 ± 14.46 years) were divided into two groups based on the presence or absence of SB: TMD_nonbruxer and TMD_bruxer. Descriptive statistics, correlation analyses, and multivariate stepwise logistic regression were conducted; *p* < 0.05 was considered statistically significant. In Cramer’s V, SB was correlated with several clinical and sleep-related factors, including TMJ noise (*r* = 0.52), TMD pain (*r* = 0.48), craniomandibular index (*r* = 0.32), limited mouth opening (*r* = 0.29), tinnitus (*r* = 0.29), an increase in the Pittsburgh sleep quality index (PSQI) global score (*r* = 0.24), and poor sleep quality, defined as a PSQI global score ≥ 5 (*r* = 0.19) (all *p* < 0.05). SB was also associated with psychological distress. Regarding hematological factors, elevated levels of cortisol (*r* = 0.30), adrenocorticotropic hormone (ACTH) (*r* = 0.34), and cortisol/ACTH ratio (*r* = 0.35) were also associated with SB (all *p* < 0.05). The factors associated with an increased likelihood of SB ranked in terms of the odds ratio (OR) were: craniomandibular index (OR = 18.400, *p* = 0.006), poor sleep quality with a PSQI global score ≥ 5 (OR = 11.425, *p* = 0.027), depression (OR = 1.189, *p* = 0.014), cortisol/ACTH ratio (OR = 1.151, *p* = 0.007), anxiety (OR = 1.081, *p* = 0.040), and adrenocorticotropic hormone (OR = 1.073, *p* = 0.019). Notably, an increase in age was associated with a decreased likelihood of SB (OR = 0.905, *p* = 0.006), with a cut-off value of 50 years (AUC = 0.259, 95% CI: 0.149–0.368, *p* = 0.024), indicating a significant decrease in bruxism occurrence in individuals aged ≥ 50 years. Further analysis revealed complex interconnections between SB and its predictors. In conclusion, SB in TMD patients was associated with age < 50 years, various clinical factors, such as TMD pain and TMJ noise, poor sleep quality, psychological deterioration, and elevated cortisol and ACTH levels.

## Introduction

Temporomandibular disorder (TMD) is an umbrella term that encompasses pain and dysfunction of the temporomandibular joint (TMJ), masticatory muscles, and adjacent structures. TMD is one of the most common causes of orofacial pain of nonodontogenic origin and has a highly complex pathophysiology^1^ Consequently, TMD easily progresses from acute to chronic pain^2^. The most common symptoms include pain around the TMJ, TMJ sounds, masticatory muscle pain, and jaw functional limitations^3^ Patients with TMD frequently experience other painful and nonpainful comorbidities, such as headaches, earaches, neck aches, tinnitus, depression, psychological distress, and sleep problems^4^ Although the reasons remain unclear, TMD is 1.5 to 3 times more common in women than in men^5,6^ TMDs are highly prevalent, affecting approximately 31% of adults and older adults and 11% of children and adolescents^7^. TMD imposes a significant economic burden on individuals and represents a substantial societal burden owing to its global impact.

The Diagnostic Criteria for Temporomandibular Disorders (DC/TMD), published in 2014, is the most widely used and reliable classification system for TMD worldwide^8^ According to the DC/TMD, TMD is an umbrella term comprising heterogeneous subtypes, including arthralgia, myalgia, local myalgia, two myofascial pain disorders, four disc displacement disorders, degenerative joint disease, subluxation, and headache attributed to TMD^3,8^ DC/TMD is based on a biopsychosocial model for understanding TMD and utilizes a dual-axis assessment tool: Axis I (physical aspects) and Axis II (psychosocial aspects). Patients with TMD are prone to psychological distress such as depression, anxiety, and somatization^9^ Additionally, there is an ongoing effort to consider environmental, genetic, and epigenetic factors in the diagnosis and treatment of patients with TMD^10^ Owing to the close relationship between persistent pain and sleep disturbance, sleep-related aspects also have been examined in patients with TMD^11,12^.

Bruxism has been defined as a repetitive jaw-muscle activity characterized by clenching or grinding of the teeth and/or bracing or thrusting of the mandible, and it is classified as either sleep bruxism (SB) or awake bruxism^13,14^ Self-reported sleep bruxism is present in approximately 12% of the adult population, with prevalence peaking between the ages of 20 and 40 and declining with increasing age^15^ SB is associated with elevated stress and somatic anxiety^16,17^, and may be linked to obstructive sleep apnea (OSA) through sleep-related arousal reactions^18^ Furthermore, the Orofacial Pain: Prospective Evaluation and Risk Assessment (OPPERA) project used a prospective cohort study design to investigate risk factors for first-onset TMD, and bruxism was found to be the strongest predictor of TMD incidence^19^Approximately 90% of patients with TMD experience sleep problems^20^, and chronic patients with TMD have higher Pittsburgh sleep quality index (PSQI) global scores compared to healthy controls^12^ In patients with TMD, individuals experiencing stress have a 2.07-fold increased likelihood of existing bruxism compared with those without stress^21^ However, Raphael et al. rebutted the hypothesis that bruxism induces or exacerbates myofascial pain^22^ SB and awake bruxism are distinct conditions^23,24^ and should be investigated separately. The influence of SB on the development of TMD and its association with various physical and psychological factors remains unclear.

Cortisol, a steroidal hormone secreted by the zona fasciculata of the adrenal glands, is a primary stress hormone in the body^25^ Adequate cortisol levels are required to maintain homeostasis, since cortisol has potent anti-inflammatory effects and is released as a terminal component of HPA-mediated stress responses^26^ The hypothalamus-pituitary-adrenal (HPA) axis regulates cortisol production and secretion. When the body perceives stimuli as threats, the hypothalamus activates the HPA axis^27^ First, corticotropin-releasing hormone (CRH) is released by the paraventricular nucleus of the hypothalamus, which then acts on the anterior pituitary to release adrenocorticotropic hormone (ACTH). ACTH acts on the adrenal cortex and regulates the rate-limiting step of cortisol synthesis. In the negative feedback loop, sufficient cortisol levels inhibit the release of both ACTH and CRH^28^ While cortisol is commonly used as a biomarker of stress and HPA axis activity, ACTH plays an upstream regulatory role and may offer additional insights into neuroendocrine dysregulation^29^. Despite being less frequently investigated in this context, ACTH measurement can enhance the understanding of hormonal influences on SB and TMD. As a response of the human endocrine system to psychological stress, sleep problems, and pain, the regulation of the HPA axis, as indicated by changes in cortisol and ACTH levels, is crucial^30,31^ In addition, increased salivary cortisol levels are associated with the presence of SB in patients with TMD^32^ Serum cortisol concentrations are significantly correlated with cortisol concentrations in saliva^33^ However, ACTH has not been investigated in relation to SB in patients with TMD.

As with other pain conditions, it is essential to investigate the clinical, sleep-related, psychological, and hematological factors associated with SB in patients with TMD and to explore the interrelationships among these factors. These characteristics, along with the associated endocrine responses, can be assessed using hematological markers. Accordingly, we directly measured the cortisol and ACTH levels in blood samples and evaluated the cortisol/ACTH ratio. Given the multifactorial nature of TMD, it was assumed that multiple variables would be intricately interrelated in patients with SB. Drawing on previous findings, we hypothesized that SB in patients with TMD would be associated with distinct clinical, psychological, sleep-related, and hematological profiles. Patients with SB were expected to exhibit more severe clinical manifestations, including higher pain intensity and a greater prevalence of TMJ pain and joint noise. With regard to sleep, poorer sleep quality–measured by the PSQI–was anticipated. From a psychological perspective, SB was hypothesized to be linked to elevated levels of distress, particularly depression and anxiety. Hematologically, increased serum concentrations of cortisol and ACTH were expected, reflecting activation of the HPA axis. Finally, an inverse association between age and the presence of SB was anticipated, with older individuals being less likely to exhibit SB.

The objectives of this cross-sectional observational study were twofold: first, to identify the differences in these factors based on the presence or absence of SB in patients with TMD; and second, to determine which factors most strongly influence the presence of SB. We hypothesized that a complex correlation exists between the occurrence of SB and these parameters in patients with TMD. Specifically, the hypothesis suggests that the presence of SB in patients with TMD can be predicted by cortisol and ACTH levels and is associated with patient age, increased TMD pain, psychological distress, and sleep disturbances. To test this hypothesis, we conducted a comprehensive investigation of the clinical, sleep-related, psychological, and hematological aspects that reflect HPA axis activation in patients with TMD.

## Methods

### Study population

The research protocol for this study was reviewed to ensure compliance with the principles of the Declaration of Helsinki and approved by the Institutional Review Board of Kyung Hee University Dental Hospital in Seoul, South Korea (KHD IRB, IRB No-KH-DT23012). Informed consent was obtained from all the participants.

### Participants

This study encompassed 79 patients with TMD pain (69 females and 10 males; mean age, 45.46 ± 14.46 years; range, 19–68 years) who presented at the Kyung Hee University Dental Hospital between January 2020 and May 2024. All patients underwent a comprehensive examination following the DC/TMD protocol, including clinical assessment, symptom questionnaires, demographic data, and an oral behavior checklist. The diagnosis of TMD was made independently by two experienced TMD specialists (YHL and QSA), each with over eight years of clinical experience. Inter-rater agreement yielded Cohen’s kappa coefficients ranging from 0.84 to 0.88, indicating strong reliability. For the evaluation of clinical and questionnaire data, intra-rater and inter-rater reliability exceeded 85% and 83%, respectively. Discrepancies were resolved through multiple consensus discussions until full agreement was reached.

The inclusion criteria were as follows: participants (1) underwent a physical examination according to the DC/TMD and aged ≥ 18 years and (2) completed the three questionnaires (PSQI, STOP-Bang, and SCL-90-R) and blood tests. The exclusion criteria were as follows: (1) systemic inflammatory connective tissue disorders, (2) history of facial tumors or surgical interventions, (3) pregnancy, (4) occult neuralgia in the orofacial region, (5) localized facial infections, (6) psychiatric disorders requiring pharmacological treatment, (7) systemic autoimmune or endocrine conditions requiring medication; and (8) deemed unsuitable for analysis due to incomplete data.

For sample size calculation, the G*Power software (latest version 3.1.9.7; Heinrich-Heine-Universität Düsseldorf, Düsseldorf, Germany) was used. A total of 42 participants (alpha error = 0.05, actual power = 0.95) were included, with a target of at least 30 participants per group deemed suitable for statistical analysis, ultimately resulting in the recruitment of 79 participants. The patients with TMD were stratified into two groups based on the presence or absence of SB: Group 1 comprised 42 patients with TMD without SB (TMD_nonbruxers) (35 females and 7 males; mean age, 50.81 ± 12.72 years), and Group 2 comprised 30 patients with TMD with concurrent SB (TMD_bruxers) (34 females and 3 males; mean age, 39.38 ± 14.04 years).

## Study design

### Clinical evaluation

#### Demographics and clinical characteristics of TMD

Patient demographics, including age, sex, height, body weight, body mass index (BMI, weight/height^2^), and neck circumference, were collected. BMI ≥ 25 kg/m² was the threshold for being overweight^34^ The duration of pain due to TMD was reported in months. When the symptom duration was > 6 months (180 days), it was regarded as having ‘chronicity’ of TMD^35^ TMD pain was scored subjectively by the patients, ranging from 0 (no pain at all) to 10 (the worst pain imaginable), using an NRS. TMD pain refers to pain occurring in the TMJ and adjacent structures during movement or at rest and is associated with functional impairment or inflammatory conditions of the TMJ and masticatory muscles. The Craniomandibular Index (CMI) was calculated based on an assessment of TMJ and mandibular dysfunction, as well as the palpation-based evaluation of masticatory muscle tenderness, following the original scoring protocol without modification^36,37^. The CMI ranges from 0 to 1, with values closer to 1 indicating higher symptom severity.

The chief complaints of patients with TMD were classified into three categories: TMJ noise, TMD pain, and LMO. The chief complaints were organized into one, two, or multiple complaints based on patient reports. TMJ noise was considered present when clicking, fine, and/or coarse crepitus, and popping noises in the TMJ occurred during mandibular movement^36^ TMD pain included pain around the TMJ, muscles, ears, and temple areas^38^LMO was defined as a comfortable mouth opening < 35 mm^8^, leading to difficulty or discomfort when opening the mouth.

####  Evaluation of sleep bruxism

To evaluate the presence of SB, participants were asked to self-report symptoms, such as audible teeth grinding during sleep, transient headaches related to nocturnal bruxism, jaw muscle pain, and muscle fatigue. SB was diagnosed if the patient responded “yes” to at least two of the following questions, with the first question being mandatory^39,40^:

1. Are you aware or have anyone mentioned that you frequently grind your teeth during sleep?
2. Do you notice that your teeth appear more worn down than expected?
3. Do you experience TMD symptoms, such as jaw fatigue, tightness, or soreness, upon awakening?
4. Do you wake up with a sensation of clenched teeth or a sore mouth?
5. Do you experience aching in your temples upon awakening?

Additionally, a visual examination was conducted to detect signs of tooth wear indicative of abnormal bruxism. In line with the Standardized Tool for the Assessment of Bruxism (STAB) criteria, the diagnosis of SB in this study corresponds to “possible sleep bruxism,” as it is based on self-report and clinical signs without polysomnographic confirmation^41^. For clarity and consistency, this condition is referred to as “SB” throughout the manuscript. To minimize overlap with awake bruxism, participants were instructed to report symptoms specifically experienced during or immediately after sleep, rather than those occurring during daytime activities or periods of wakefulness.

#### Contributing factors or comorbidities for TMD

The presence of psychological stress was evaluated using the dichotomous question ‘Have you encountered any mental stress or psychological pressure in your daily life over the past week?’ Additionally, the participants were asked about their sleep problems and tinnitus. All variables were recorded in binary format (yes/no) for all patients, following the methodology detailed in our previous study^42^.

#### Sleep quality evaluation using PSQI

The PSQI, a self-rated sleep questionnaire, was used to evaluate the sleep quality. Habitual sleep quality and sleep disturbances over the past month were assessed using the 19-item PSQI. The PSQI comprises seven components that address subjective sleep quality, sleep latency, sleep duration, sleep efficiency, sleep disturbances, use of sleep medication, and daytime dysfunction. Each subscale is equally weighted and scored from 0 (good sleep/no problems) to 3 (poor sleep/severe problems), culminating in a global PSQI score ranging from 0 to 21. Higher scores denote poorer sleep quality, and a global score higher than 5 has diagnostic value for distinguishing poor from good sleep^43^.

#### Risk evaluation of OSA with STOP-Bang

The STOP-BANG questionnaire is a validated screening tool for identifying individuals with a high likelihood of OSA. This instrument comprises eight dichotomous (yes/no) questions related to the clinical features of sleep apnea. Each “yes” response scores 1 point, while a “no” response scores 0, with the total score ranging from 0 to 8. A high likelihood for OSA was present when ≥ 3 questions were answered as “yes”^44^.

#### Psychological aspects with SCL-90-R

The psychological status of the participants was investigated using the SCL-90-R^45^ Patients responded to 90 questions on a five-point Likert scale ranging from 0 (not at all) to 4 (extremely), specifying the extent to which each item had affected them within the past seven days. This scale measures the symptom intensity and evaluates nine psychological symptom dimensions: SOM, obsessive-compulsiveness (O-C), I-S, DEP, ANX, HOS, PHOB, PAR, and psychosis PSY.

#### Hematological parameters

Blood sampling was conducted between 9:00 am and 11:00 am to minimize variability due to circadian rhythms. The test included a complete blood count, differential leukocyte counts, and various hematological variables. The levels of gonadal hormones, including stress markers such as cortisol, ACTH, and CAR were measured. Additionally, the ESR was measured in blood samples. Reference ranges for the variables were as follows: cortisol (morning): 5–27 µg/dL, ACTH: 10–60 pg/mL, and ESR: reference range: 0–20 mm/h^46,47^.

#### Reliability and measurement error

Inter- and intra-observer reliabilities were assessed to determine the degree of agreement. All parameters and TMD diagnoses based on the DC/TMD criteria were separately confirmed by two investigators (YHL and QSA). Interclass correlation coefficients (ICCs; 0–1 (no reliability to perfect reliability)^48^ were calculated with a prespecified threshold that correlated between assessments and exceeded 0.80 for all items. In instances of disagreement, a unified conclusion was reached through extensive discussions until a consensus was reached. With repeated assessments, the ICC consistently met the criterion (> 0.80) in all the cases.

### Statistical analysis

Data were analyzed using the Statistical Package for Social Sciences (SPSS) for Windows (version 26.0; IBM Corp., Armonk, NY, USA). Descriptive statistics were presented as mean ± standard deviation or as frequencies with percentages, as appropriate. To analyze the distribution of categorical data, we employed the χ^2^ test and Bonferroni correction for equality of proportions. The t-test was used to compare the mean values between the two groups. Spearman’s correlation analysis was conducted to evaluate the strength of the association between two variables. Cramer’s V analysis was used to assess the strength of the association between two variables. Cramer’s V value ranges from 0 to 1, with values closer to 1 indicating a stronger correlation between the variables^49^. ROC curves were plotted and the corresponding AUC values were calculated to assess the performance of the models at the classification threshold (above the mean value of each laboratory parameter). The revised classification is as follows: AUC = 0.5 (no discrimination), 0.5 < AUC ≤ 0.7 (poor discrimination), 0.7 < AUC ≤ 0.8 (acceptable discrimination), 0.8 < AUC ≤ 0.9 (excellent discrimination), and AUC > 0.9 (outstanding discrimination)^50^. Multivariate stepwise logistic regression analysis was performed to evaluate risk factors for SB. Parameters that correlated with SB were simultaneously considered to calculate the OR for a high likelihood of SB (the dependent variable). R (version 4.0.2; R Foundation for Statistical Computing, Vienna, Austria) was used to derive the hierarchy and clusters of correlations. A two-tailed *p*-value of less than 0.05 was considered statistically significant for all analyses.

## Results

### Demographics

The overall female-to-male ratio was 6.9:1, indicating a significantly higher proportion of females in the study sample. While the general prevalence of TMD is commonly reported to be approximately 2:1 (female to male), treatment-seeking behavior has been observed at ratios ranging from 3:1 to as high as 9:1^51,52^. The predominance of female participants in this study might reflect both the higher prevalence of TMD in females and a greater willingness among female patients to consent to blood-based research procedures. The ratios in the TMD_nonbruxer and TMD_bruxer groups were 4.99:1 and 11.35:1 (*p* = 0.322), respectively. The mean age of the TMD_bruxer group (39.38 ± 14.04 years) was significantly lower than that of the TMD_nonbruxer group (50.81 ± 12.72 years) (*p* < 0.001) (Table 1).

**Table 1:** Clinical, sleep, psychological, and hematological characteristics.

Parameters	TMD_Nonbruxer (*n* = 42)	TMD_Bruxer (*n* = 37)	*p*-value
	mean ± SD or *n* (%)	mean ± SD or *n* (%)	
Demographics			
Sex (Female) ^a^	35 (83.3%)	34 (91.9%)	0.322
Age (Years) ^b^	50.81 ± 12.72	39.38 ± 14.04	< 0.001***
BMI (kg/m^2^) ^b^	21.24 ± 3.48	22.38 ± 3.40	0.144
Clinical characteristics			
Symptom duration (Months) ^b^	31.02 ± 46.32	32.67 ± 57.74	0.888
Chronicity ^a^	24 (57.1%)	27 (73.0%)	0.164
TMJ noise ^a^	9 (21.4%)	29 (78.4%)	< 0.001***
TMD pain ^a^	3 (7.1%)	18 (48.6%)	< 0.001***
Pain intensity (NRS) ^b^	4.36 ± 2.60	3.68 ± 2.82	0.267
Pain intensity (CMI) ^b^	0.11 ± 0.16	0.23 ± 0.21	0.005**
LMO ^a^	6 (14.3%)	15 (40.5%)	0.011*
Tinnitus ^a^	2 (4.8%)	8 (21.6%)	0.039*
Sleep characteristics			
Sleep problem ^a^	10 (23.8%)	19 (51.4%)	0.019*
Sleep Quality (PSQI global score) ^b^	7.88 ± 4.60	10.38 ± 3.61	0.009**
Poor sleeper ^a^	27 (64.3%)	32 (86.5%)	0.037*
OSA (STOP-Bang score) ^b^	2.26 ± 1.45	2.49 ± 1.41	0.488
OSA (High risk of OSA) ^a^	16 (38.1%)	18 (48.6%)	0.371
Psychological characteristics			
Psychological stress ^a^	9 (21.4%)	17 (45.9%)	0.030*
SOM ^b^	48.98 ± 12.33	49.73 ± 9.73	0.766
O-C ^b^	41.43 ± 9.88	42.81 ± 9.53	0.530
I-S ^b^	43.47 ± 10.68	49.03 ± 8.65	0.014*
DEP ^b^	43.45 ± 10.32	48.59 ± 9.65	0.026*
ANX ^b^	43.72 ± 9.65	50.94 ± 13.47	0.003**
HOS ^b^	43.98 ± 8.026	45.78 ± 7.28	0.300
PHOB ^b^	46.19 ± 11.19	48.11 ± 10.29	0.433
PAR ^b^	45.26 ± 14.55	43.59 ± 8.75	0.546
PSY ^b^	45.76 ± 11.98	46.89 ± 9.87	0.651
Hematological factors			
Cortisol (ug/dL) ^b^	4.98 ± 5.05	7.64 ± 10.47	0.022*
ACTH (pg/mL) ^b^	21.39 ± 22.69	37.23 ± 21.49	0.004**
CAR ^b^	13.12 ± 13.54	24.78 ± 26.29	0.014*
ESR (mm/h) ^b^	18.95 ± 13.04	22.32 ± 20.88	0.386

^a^ Results were obtained using a χ^2^ test between two age groups. ^b^ Results were analyzed using a t-test. To obtain significant results, the two-tailed level of statistical significance was set at *p* < 0.05. *p < 0.05, **p < 0.01, ***p < 0.001.* OR* odds ratio,* CI* confidence interval,* LMO* limited mouth opening,* CMI* craniomandibular index,* TMJ* temporomandibular joint,* TMD* temporomandibular disorder,* ACTH*: adrenocorticotropic hormone,* CAR* cortisol/ACTH ratio,* ESR* erythrocyte sedimentation rate,* PSQI* Pittsburgh sleep quality index,* STOP-Bang* S stands for snore, T tired, O observed apneas, P pressure (arterial hypertension), B BMI (body mass index > 35 kg/ m^2^), a age (> 50 years old), n neck circumference (> 40 cm), and g gender (male);*BMI * body mass index,* in*.: inch,* OSA* obstructive sleep apnea,* SCL-90-R* symptom checklist 90-revised,* SOM* somatization,* O-C* obsessive-compulsive,* I-S* interpersonal sensitivity,* DEP* depression,* ANX* anxiety,* HOS* hostility,* PHOB* phobic anxiety,* PAR* paranoid ideation,* PSY* psychoticism.

### Clinical symptoms and TMD pain characteristics

There was no significant difference in the duration of TMD symptoms between the TMD_nonbruxer and TMD_bruxer groups (31.02 ± 46.32 vs. 32.67 ± 57.74 months, *p* = 0.888). Furthermore, a higher percentage of chronic TMD symptoms was observed in the TMD_bruxer group (73.0%) than that in the TMD_nonbruxer group (57.1%), although this difference was not statistically significant (*p* = 0.164). TMJ noise (78.4% vs. 21.4%, *p* < 0.001), TMD pain (48.6% vs. 7.1%, *p* < 0.001), and limited mouth opening (LMO; 40.5% vs. 14.3%, *p* = 0.008) were significantly more prevalent in the TMD_bruxer group than in the TMD_nonbruxer group. Furthermore, the two groups showed statistically significant differences in TMD pain intensity. The CMI (0.11 ± 0.16 vs. 0.23 ± 0.21, *p* = 0.005) was significantly higher in the TMD_bruxer group than that in the TMD_nonbruxer group. When assessing patient-reported pain using the numeric rating scale (NRS), no significant difference in TMD pain intensity was observed between the TMD_nonbruxer and TMD_bruxer groups (4.36 ± 2.60 vs. 3.68 ± 2.82, *p* = 0.267) (Table 1).

### Contributing factors for TMD with sleep bruxism

Considering the contributing factors of TMD, sleep problems (51.4% vs. 23.8%, *p* = 0.019), psychological stress (45.9% vs. 21.4%, *p* = 0.030), and tinnitus (21.6% vs. 4.8%, *p* = 0.039) were significantly more prevalent in the TMD_bruxer group than in the TMD_nonbruxer group (Table 1). Thus, the prevalence of these factors was significantly higher in patients with TMD and bruxism than in those without SB.

### Sleep quality and poor sleepers in TMD with sleep bruxism

The TMD_bruxer group showed significantly higher mean scores than those of the TMD_nonbruxer group in PSQI components 1 (subjective sleep quality: 1.73 ± 0.56 vs. 1.28 ± 0.81, *p* = 0.006), 3 (sleep duration: 1.76 ± 0.68 vs. 1.31 ± 0.87, *p* = 0.014), 4 (sleep efficiency: 1.65 ± 0.75 vs. 1.21 ± 0.87, *p* = 0.021), and 7 (daytime dysfunction: 1.43 ± 0.76 vs. 1.02 ± 0.81, *p* = 0.025). This indicates that the subjective sleep quality, sleep duration, sleep efficiency, and daytime dysfunction were more compromised and distorted in patients with TMD and bruxism than in those without bruxism (data not shown). Additionally, the PSQI global score was significantly higher in the TMD_bruxer group than that in the TMD_nonbruxer group (10.38 ± 3.61 vs. 7.88 ± 4.60, *p* = 0.009). Thus, TMD_bruxers had poorer sleep quality than TMD_nonbruxers. The proportion of poor sleepers was also significantly higher in the TMD_bruxer group (86.5%) than that in the TMD_nonbruxer group (64.3%; *p* = 0.037) (Table 1).

### High risk of OSA in TMD with sleep bruxism

Among the eight items of STOP-Bang, “tired” (70.3% vs. 33.3%, *p* = 0.002) and “age over 50” (61.9% vs. 27.0%, *p* = 0.003) were significantly higher in the TMD_bruxer group than in the TMD_nonbruxer group. However, other factors such as snoring, observed apnea, and high blood pressure did not differ significantly between the two groups (data not shown). Furthermore, the STOP-Bang total score did not show a statistically significant difference based on the absence or presence of bruxism in patients with TMD (2.26 ± 1.45 vs. 2.49 ± 1.41, *p* = 0.488). Assuming that a STOP-Bang score of 3 or higher indicates a ‘high risk of OSA,’ and no significant difference was observed between the TMD_nonbruxer (38.1%) and TMD_bruxer (48.6%) groups (*p* = 0.371) (Table 1).

### Psychological aspects in TMD with sleep bruxism

When investigating psychological aspects using the SCL-90-R, TMD_bruxer patients had significantly higher T scores compared to those of TMD_nonbruxer patients in interpersonal sensitivity (I-S: 49.03 ± 8.65 vs. 43.47 ± 10.68, *p* = 0.014), depression (DEP: 48.59 ± 9.65 vs. 43.45 ± 10.32, *p* = 0.026), and anxiety (ANX: 50.94 ± 13.47 vs. 43.72 ± 9.65, *p* = 0.003). This indicates that interpersonal sensitivity, depression, and anxiety levels were higher in patients with TMD with bruxism than in those without bruxism. The remaining six SCL-90-R parameters (SOM, O-C, HOS, PHOB, PAR, and PSY) were not significantly different between the two groups (all *p* > 0.05) (Table 1).

### Hematological characteristics of TMD with sleep bruxism

We investigated the blood test parameters related to stress and inflammatory responses in patients with TMD and bruxism. The cortisol level was significantly higher in the TMD_bruxer group than that in the TMD_nonbruxer group (7.64 ± 10.47 vs. 4.98 ± 5.05 µg/dL, *p* = 0.022). No patients had cortisol levels outside the normal reference range (5–27 µg/dL). ACTH was also significantly higher in the TMD_bruxer group (37.23 ± 21.49 vs. 21.39 ± 22.69 pg/mL, *p* = 0.004). The cortisol/ACTH ratio (CAR) was significantly higher in the TMD_bruxer group than that in the TMD_nonbruxer group (24.78 ± 26.29 vs. 13.12 ± 13.54, *p* = 0.014). The mean erythrocyte sedimentation rate (ESR), a representative marker of systemic inflammatory activity, was higher in the TMD_bruxer group (22.32 ± 20.88 mm/h) than that in the TMD_nonbruxer group (18.95 ± 13.04 mm/h), but this difference was not statistically significant (*p* = 0.386) (Table 1).

### Parameters associated with sleep bruxism in patients with TMD

**Fig. 1:**
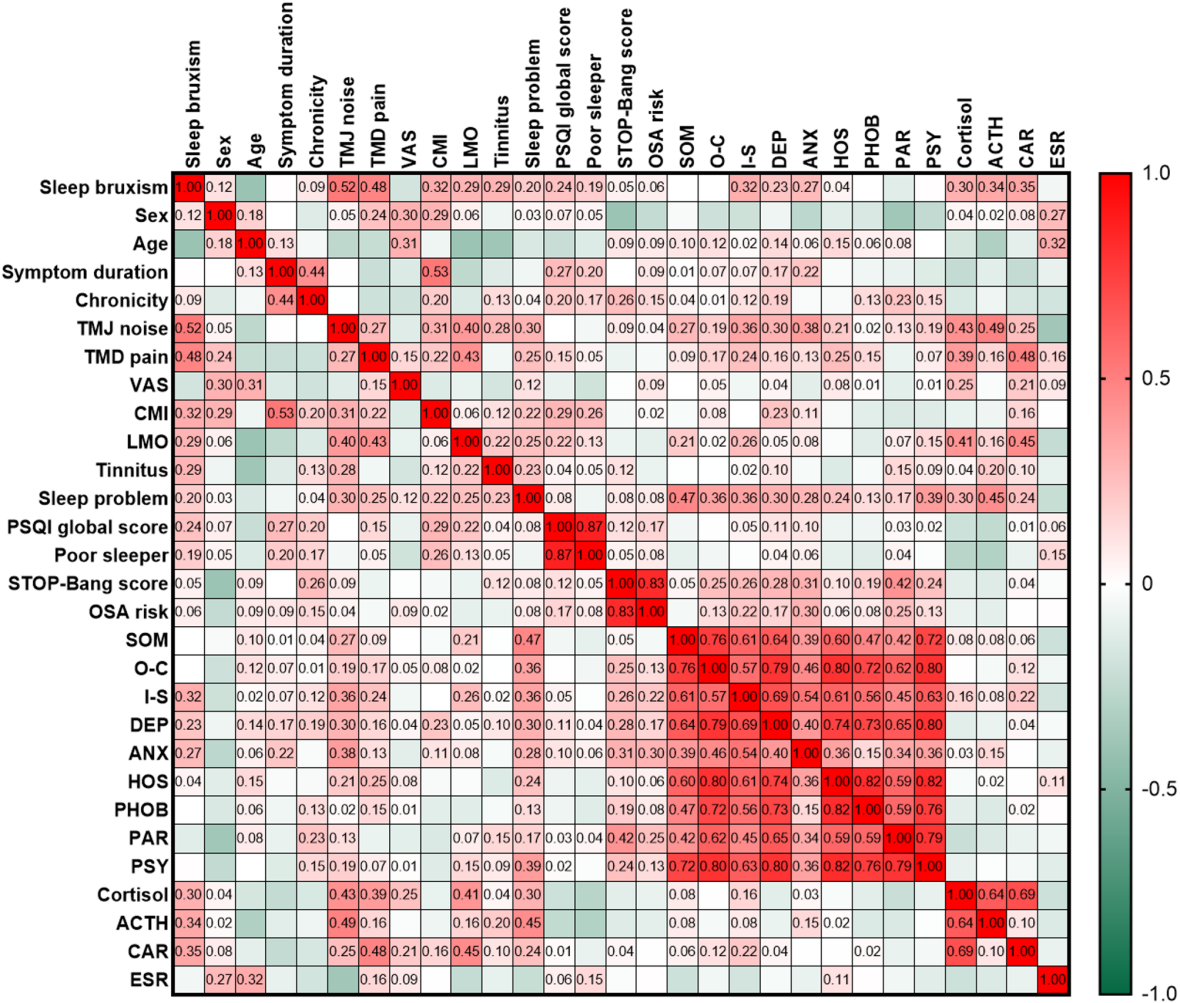
Heatmap illustrating the relationships among sleep bruxism, clinical characteristics, sleep-related parameters, psychological distress, and hematological factors in all patients with TMD. The results were derived from Cramer’s V analysis.* TMJ* temporomandibular joint,* TMD* temporomandibular disorder,* NRS* numeric rating scale,* CMI* craniomandibular index,* LMO* limited mouth opening,* PSQI* pittsburgh sleep quality index,* STOP-Bang* S stands for snore, T tired, O observed apneas, P pressure (arterial hypertension), B BMI (body mass index > 35 kg/ m^2^),* a* age (> 50 years old),* n *neck circumference (> 40 cm), and* g *gender (male), OSA: obstructive sleep apnea,* SOM* somatization,* O-C* obsessive-compulsive,* I-S* interpersonal sensitivity,* DEP* depression,* ANX* anxiety,* HOS* hostility,* PHOB* phobic anxiety,* PAR* paranoid ideation,* PSY* psychoticism,* ACTH* adrenocorticotropic hormone,* CAR* cortisol/ACTH ratio,* ESR* erythrocyte sedimentation rate.

Figure 1 illustrates the factors associated with bruxism among the various aspects investigated in patients with TMD as well as the strength of these correlations (r). In this study, SB was associated with several clinical factors including TMD pain, TMJ noise, CMI, LMO, and tinnitus. Sleep-related factors were significantly associated with an increase in the PSQI global score and the presence of poor sleepers. Additionally, SB was associated with psychological distress. Increased cortisol, ACTH, and CAR levels were also associated with the presence of SB. Complex interconnections were also observed among these factors.

In patients with TMD, the clinical parameter most strongly positively correlated with SB was TMJ noise (*r* = 0.52), followed by TMD pain (*r* = 0.48), pain intensity (CMI) (*r* = 0.32), LMO (*r* = 0.29), and tinnitus (*r* = 0.29). Among the sleep-related parameters, the PSQI global score (*r* = 0.24), presence of self-reported sleep problems (*r* = 0.20), and classification as poor sleeper (*r* = 0.19) were correlated with SB. In terms of psychological distress, interpersonal sensitivity (I-S; *r* = 0.32), ANX (*r* = 0.27), and depression (DEP; *r* = 0.23) scores were correlated with the presence of SB in patients with TMD. Regarding hematological factors, cortisol (*r* = 0.30), ACTH (*r* = 0.34), and CAR (*r* = 0.35) were associated with the presence of SB in patients with TMD (all *p* < 0.05).

Furthermore, there were several complex intercorrelations among the clinical, sleep-related, psychological, and hematological factors. One of the key relationships observed was that the presence of TMD pain was significantly correlated with the CAR (*r* = 0.49) and cortisol levels (*r* = 0.39) (all *p* < 0.05). The presence of self-reported sleep problems was significantly correlated with hematological cortisol (*r* = 0.30) and ACTH (*r* = 0.45) levels. Sleep problems were also correlated with the presence of TMD pain (*r* = 0.25), pain intensity as measured by the NRS (*r* = 0.12), and scores on the nine psychological distress parameters of the SCL-90R (somatization (SOM): *r* = 0.47, O-C: *r* = 0.36, I-S: *r* = 0.36, DEP: *r* = 0.30, ANX: *r* = 0.28, hostility (HOS): *r* = 0.24, phobic anxiety (PHOB): *r* = 0.13, paranoid ideation (PAR): *r* = 0.17, psychoticism (PSY): *r* = 0.39). The chronicity of TMD symptoms was significantly associated with cortisol (*r* = 0.43), ACTH (*r* = 0.40), and CAR (*r* = 0.25) levels. The STOP-Bang score (*r* = 0.26) and the presence of OSA risk, indicated by a STOP-Bang total score ≥ 3 (*r* = 0.15), were significantly correlated with the chronicity of TMD symptoms. Additionally, increasing age was correlated with higher subjective pain intensity as measured by the NRS (*r* = 0.31) (all *p* < 0.05).

### Hierarchical relationships and interrelations

**Fig. 2:**
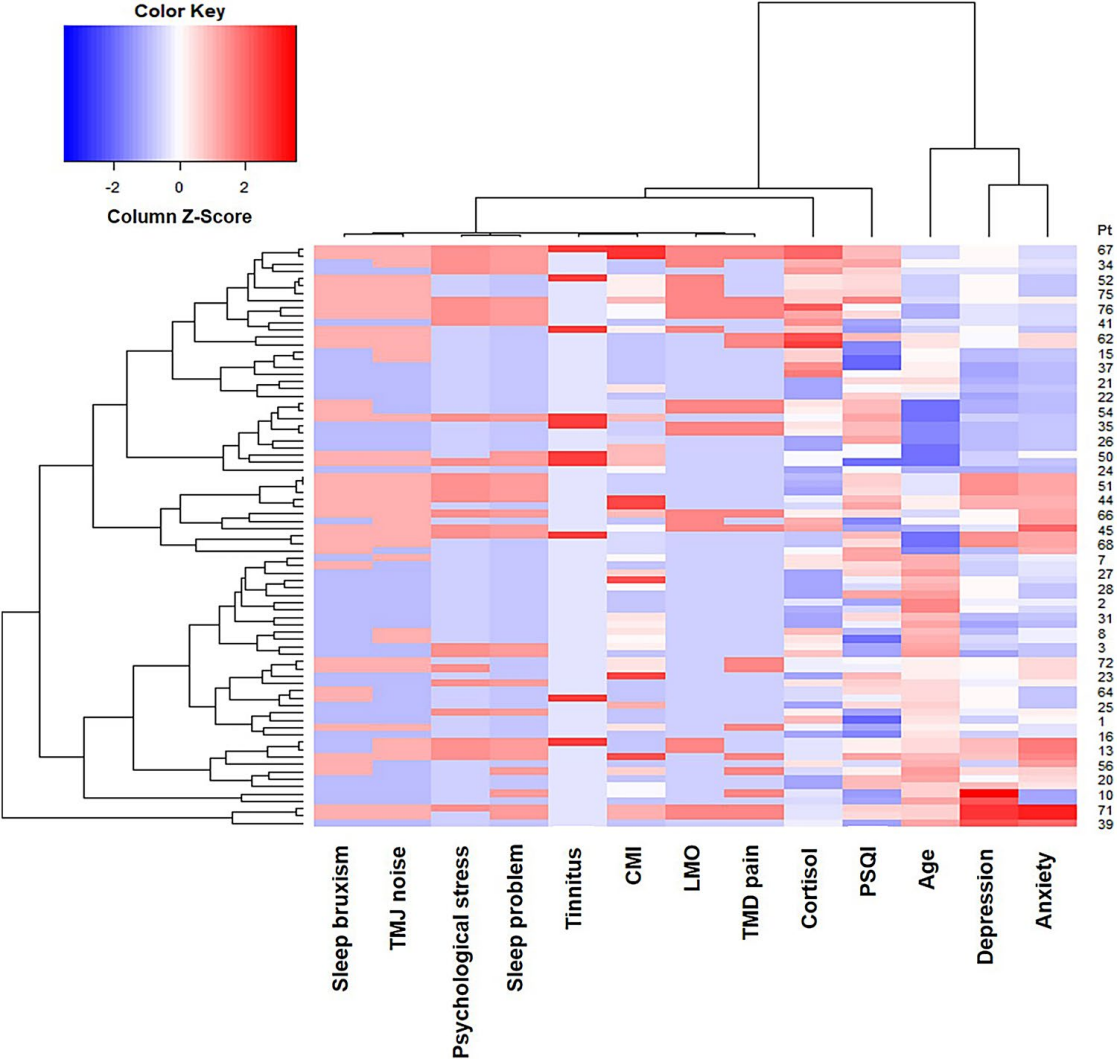
Hierarchy and clusters of key correlations. Correlations between the parameters and clusters with complex relationships were obtained using R (version 4.0.2).* CMI* craniomandibular index,* LMO* limited mouth opening,* PSQI* Pittsburgh sleep quality index global score.

Using R, we examined the complex hierarchical relationships and interrelations among the groups of parameters. At the first level, SB was correlated with TMJ noise, psychological stress was correlated with sleep problems, and tinnitus was correlated with the CMI score and LMO. These clusters were subsequently found to be correlated with the cortisol levels at the second level. At the third level, these parameters were associated with the PSQI. Additionally, DEP levels assessed using the SCL-90R correlated with ANX levels, and these were further associated with aging at the second level. These two major clusters were interrelated (Fig. 2).

### Single logistic regression analysis for SB

To identify the significant predictors of SB in patients with TMD, a single logistic regression analysis was performed for each variable (Table 2). First, considering the clinical characteristics, the presence of TMJ noise significantly increased the likelihood of SB in patients with TMD by 13.292 times (odds ratio (OR) = 13.292, 95% confidence interval (CI): 0.456–38.949, *p* < 0.001). TMD pain increased the risk of SB by 12.316 times (OR = 12.316, 95% CI, 3.226–47.017; *p* < 0.001). The presence of LMO and tinnitus increased the likelihood of SB by 4.091 times (OR = 4.091, 95% CI: 1.382–12.109, *p* = 0.011) and 5.517 times (OR = 5.517, 95% CI: 1.090–27.922, *p* = 0.039), respectively. Among the pain intensity measures, the objective index CMI increased the risk of SB by 2.624 times (OR = 2.624, 95% CI: 1.046–6.548, *p* = 0.040). Secondly, among sleep-related parameters, an increase in the PSQI global score (OR = 3.556, 95% CI: 1.032–1.299, *p* = 0.013) and being classified as a poor sleeper based on the PSQI (OR = 3.556, 95% CI: 1.144–11.055, *p* = 0.028) were associated with an increased likelihood of SB. The STOP-Bang score was not a significant predictor of SB in patients with TMD. Third, from a psychological perspective, increases in I-S (OR = 1.060, 95% CI: 1.010–1.112, *p* = 0.018), DEP (OR = 1.055, 95% CI: 1.004–1.108, *p* = 0.033), and ANX (OR = 1.062, 95% CI: 1.017–1.109, *p* = 0.006) were associated with a slight but significant increase in the likelihood of SB by 1.060-, 1.055, and 1.062 times, respectively. With regard to hematological factors, increases in cortisol (OR = 1.099, 95% CI: 1.003–1.205, *p* = 0.043), ACTH (OR = 1.032, 95% CI: 1.009–1.056, *p* = 0.006), and CAR (OR = 1.035, 95% CI: 1.004–1.066, *p* = 0.025) were associated with a slight but significant increase in the risk of SB by 1.099, 1.032, and 1.035 times, respectively. Interestingly, an increase in age was significantly associated with a decreased likelihood of SB, with the likelihood decreasing 0.939 times for each year of age increase (OR = 0.939, 95% CI: 0.905–0.975, *p* = 0.001).

**Table 2:** Logistic regression analysis for predicting sleep bruxism.

Parameters	Analysis 1: Single logistic regression analysis				Analysis 2: Multiple logistic regression analysis with backward selection			
	OR	Lower 95% CI	Upper 95% CI	*p*-value	OR	Lower 95% CI	Upper 95% CI	*p*-value
Demographics								
Sex (Female)	2.267	0.541	9.495	0.263				
Age (Years)	**0.939**	**0.905**	**0.975**	0.001**	**0.905**	**0.842**	**0.972**	0.006**
BMI (kg/m^2^)	1.106	0.964	1.270	0.152				
Clinical characteristics								
Symptom duration (Months)	1.001	0.992	1.009	0.887				
Chronicity	2.025	0.784	5.229	0.145				
TMJ noise	**13.292**	**4.536**	**38.949**	< 0.001***				
TMD pain	**12.316**	**3.226**	**47.017**	< 0.001***				
Pain intensity (NRS)	0.909	0.769	1.075	0.264				
Pain intensity (CMI)	**2.624**	**1.046**	**6.584**	0.040*	**18.400**	**2.323**	**145.731**	0.006**
LMO	**4.091**	**1.382**	**12.109**	0.011*				
Tinnitus	**5.517**	**1.090**	**27.922**	0.039*				
Sleep characteristics								
Sleep problem	**3.378**	**1.295**	**8.813**	0.013*				
Sleep Quality (PSQI global score)	**1.158**	**1.032**	**1.299**	0.013*				
Poor sleeper	**3.556**	**1.144**	**11.055**	0.028*	**11.425**	**1.327**	**98.364**	0.027*
OSA (STOP-Bang score)	1.119	0.817	1.532	0.483				
OSA (High risk of OSA)	1.539	0.628	3.773	0.345				
Psychological characteristics								
Psychological stress	**3.117**	**1.169**	**8.308**	0.023*				
SOM	1.006	0.967	1.047	0.763				
O-C	1.015	0.969	1.063	0.525				
I-S	**1.060**	**1.010**	**1.112**	0.018*				
DEP	**1.055**	**1.004**	**1.108**	0.033*	**1.189**	**1.035**	**1.365**	0.014*
ANX	**1.062**	**1.017**	**1.109**	0.006**	**1.081**	**1.004**	**1.164**	0.040*
HOS	1.032	0.972	1.097	0.303				
PHOB	1.017	0.975	1.061	0.433				
PAR	0.988	0.952	1.026	0.543				
PSY	1.010	0.969	1.051	0.647				
Hematological factors								
Cortisol (ug/dL)	**1.099**	**1.003**	**1.205**	0.043*				
ACTH (pg/mL)	**1.032**	**1.009**	**1.056**	0.006**	**1.073**	**1.011**	**1.139**	0.019*
CAR	**1.035**	**1.004**	**1.066**	0.025*	**1.151**	**1.040**	**1.275**	0.007**
ESR (mm/h)	1.012	0.986	1.039	0.383				

To obtain significant results, the two-tailed level of statistical significance was set at *p* < 0.05. *p < 0.05, **p < 0.01, ***p < 0.001. * OR* odds ratio,* CI* confidence interval,* LMO* limited mouth opening,* CMI* craniomandibular index,* TMJ* temporomandibular joint,* TMD* temporomandibular disorder,* PSQI* Pittsburgh sleep quality index,* STOP-Bang* S stands for snore, T tired, O observed apneas, P pressure (arterial hypertension), B BMI (body mass index > 35 kg/ m^2^), a age (> 50 years old), n neck circumference (> 40 cm), and g gender (male); *BMI * body mass index,* in*.: inch,* OSA* obstructive sleep apnea,* SCL-90-R* symptom checklist 90-revised,* SOM* somatization,* O-C* obsessive-compulsive,* I-S* interpersonal sensitivity,* DEP* depression,* ANX* anxiety,* HOS* hostility,* PHOB* phobic anxiety,* PAR* paranoid ideation,* PSY* psychoticism.* ACTH* adrenocorticotropic hormone,* CAR:*cortisol/ACTH ratio,* ESR* erythrocyte sedimentation rate

### Multiple logistic regression analysis for SB

To further investigate which factors significantly predicted the presence of SB in patients with TMD in a comprehensive and multifactorial manner, a multiple logistic regression analysis was performed (Table 2). The factor most strongly associated with an increased likelihood of SB was CMI, an objective measure of pain intensity; a CMI value above the mean increased the likelihood of SB by 18.4 times (OR = 18.400, 95% CI: 2.323–145.731, *p* = 0.006). Next, being a poor sleeper, based on a PSQI global score of 5 or higher, increased the likelihood of SB by 11.425 times (OR = 11.425, 95% CI: 1.327–98.364, *p* = 0.027). Among the psychological factors, increases in DEP (OR = 1.189, 95% CI: 1.035–1.365, *p* = 0.014) and ANX (OR = 1.081, 95% CI: 1.004–1.164, *p* = 0.040) were associated with a slight but significant increase in the risk of SB by 1.189 and 1.081 times, respectively. Among the hematological factors, increases in ACTH (OR = 1.073, 95% CI: 1.011–1.139, *p* = 0.019) and CAR (OR = 1.151, 95% CI: 1.040–1.275, *p* = 0.007) were associated with an increased risk of SB by 1.073 and 1.151 times, respectively. Notably, an increase in age was associated with a decreased likelihood of developing SB (OR = 0.905, 95% CI, 0.842–0.972; *P* = 0.006). These results indicate that various clinical, sleep-related, psychological, and hematological factors contribute to the likelihood of SB in patients with TMD, either by increasing or decreasing the risk based on their presence and levels.

### Cut-off value for predicting SB

**Fig. 3:**
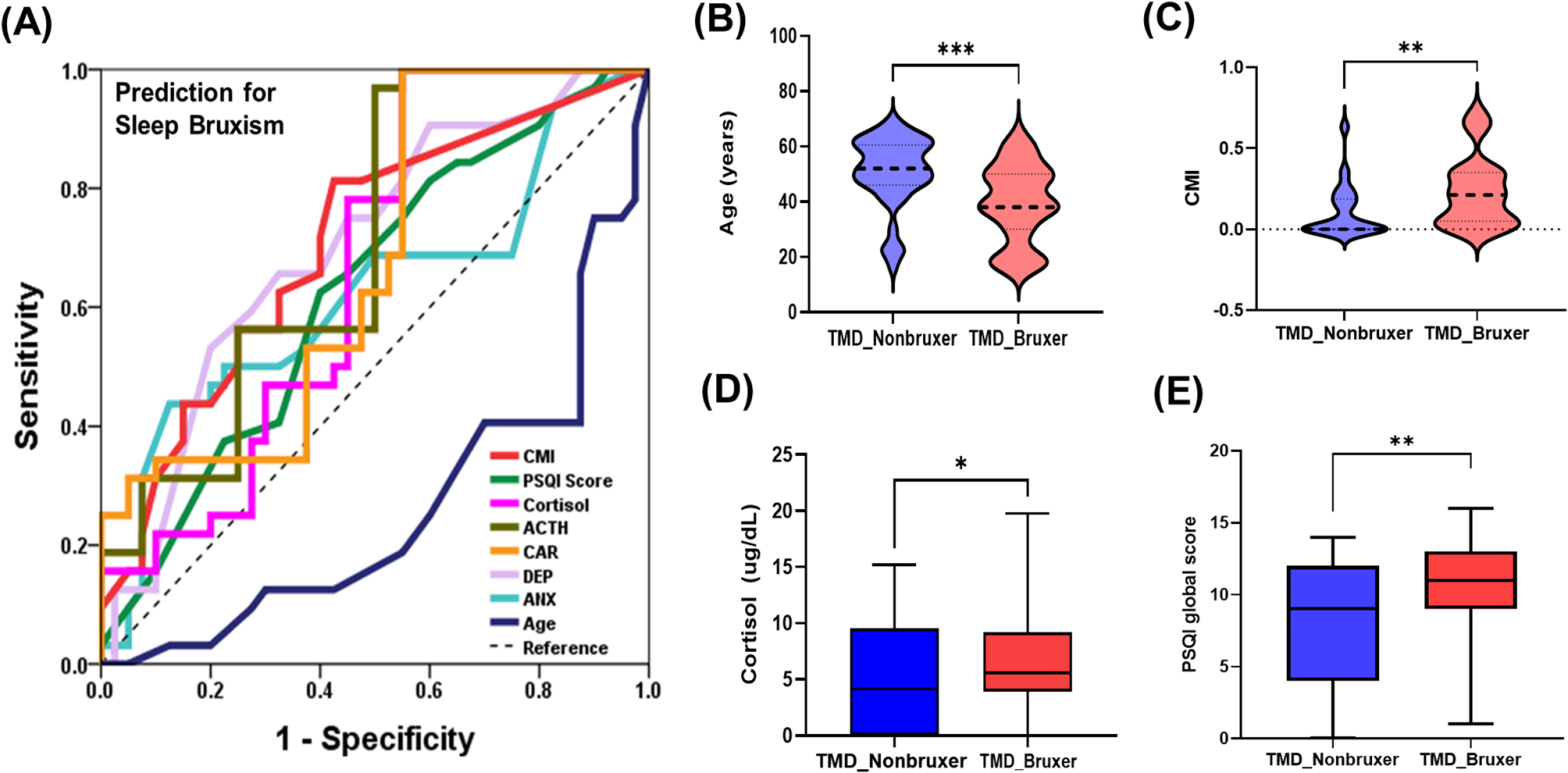
Statistical analysis of individual factors predicting sleep bruxism.(**A**) Receiver operating characteristic curve with bruxism as the outcome variable. Comparison of (**B**) age, (**C**) CMI, (**D**) cortisol, and (**E**) PSQI global scores, which are key parameters distinguishing between the TMD_nonbruxer and TMD_bruxer groups. For A, the results were obtained using the ROC analysis. Results for B, C, D, and E were analyzed using t-tests.* TMD* temporomandibular disorder,* CMI* craniomandibular index,* PSQI Score* pittsburgh sleep quality index global score,* ACTH* adrenocorticotropic hormone; cortisol/ACTH ratio,* DEP* depression,* ANX* anxiety. To obtain significant results, the two-tailed level of statistical significance of the p-value was set at *p* < 0.05. *p-value < 0.05, **p-value < 0.01, ***p-value < 0.001.

In the receiver operating characteristic (ROC) analysis, several continuous parameters significantly predicted SB in patients with TMD (Table 3). The cut-off value for predicting bruxism using the CMI was 0.143 (AUC = 0.713, 95% CI: 0.599–0.827, *p* = 0.024). When using the PSQI global score to predict bruxism, the cutoff value was 10.5 (AUC = 0.648, 95% CI: 0.527–0.770, *p* = 0.024). The cut-off value for cortisol in predicting SB was 7.105 µg/dL (AUC = 0.643, 95% CI: 0.519–0.766, *p* = 0.029). The cut-off values for ACTH and CAR in predicting SB were 28.575 pg/mL (AUC = 0.708, 95% CI: 0.588–0.828, *p* = 0.003) and 17.39 (AUC = 0.629, 95% CI: 0.504–0.754, *p* = 0.049), respectively. Considering psychological factors, the DEP and ANX scores were significant predictors of SB. The cut-off value for DEP was 49.51 (AUC = 0.706, 95% CI: 0.591–0.821), and for ANX, it was 42.54 (AUC = 0.661, 95% CI: 0.538–0.784). The cutoff value for age was 50 years (AUC = 0.259, 95% CI: 0.149–0.368, *p* = 0.024), indicating that the likelihood of bruxism decreased significantly in individuals aged 50 years and above. Based on these findings, key parameters that can distinguish between the TMD_nonbruxer and TMD_bruxer groups in patients with TMD, including CMI, PSQI global score, cortisol, ACTH, CAR, DEP, ANX, and age, are shown in Fig. 3.

**Table 3:** Cut-off values for each parameter for sleep bruxism.

Parameter	Cut-off value	AUC (standard error)	95% CI		*p*-value
			Lower	Upper	
CMI (0–1)	**0.143**	0.713 (0.058)	0.599	0.827	0.001**
PSQI global score (0–21)	**10.5**	0.648 (0.062)	0.527	0.77	0.024*
Cortisol	**7.105 ug/dL**	0.643 (0.063)	0.519	0.766	0.029*
ACTH	**28.575 pg/mL**	0.708 (0.061)	0.588	0.828	0.003**
CAR	**17.39**	0.629 (0.064)	0.504	0.754	0.049*
DEP	**49.51**	0.706 (0.059)	0.591	0.821	0.002**
ANX	**42.54**	0.661 (0.063)	0.538	0.785	0.014*
Age	**50.0 years**	0.259 (0.056)	0.149	0.368	< 0.001***

The results were obtained using AUC analysis in all patients with TMD (*n* = 79). *TMD* temporomandibular disorder,* CMI* craniomandibular index,* PSQI* Pittsburgh sleep quality index,* ACTH* adrenocorticotropic hormone,* CAR* cortisol/ACTH ratio,* DEP* depression,* ANX* anxiety,* AUC* area under the curve,* CI* confidence interval. Statistical significance was set at *p* < 0·05· *p < 0·05, **p < 0·01, ***p < 0·001. Significant results are indicated in bold.

## Discussion

**Fig. 4:**
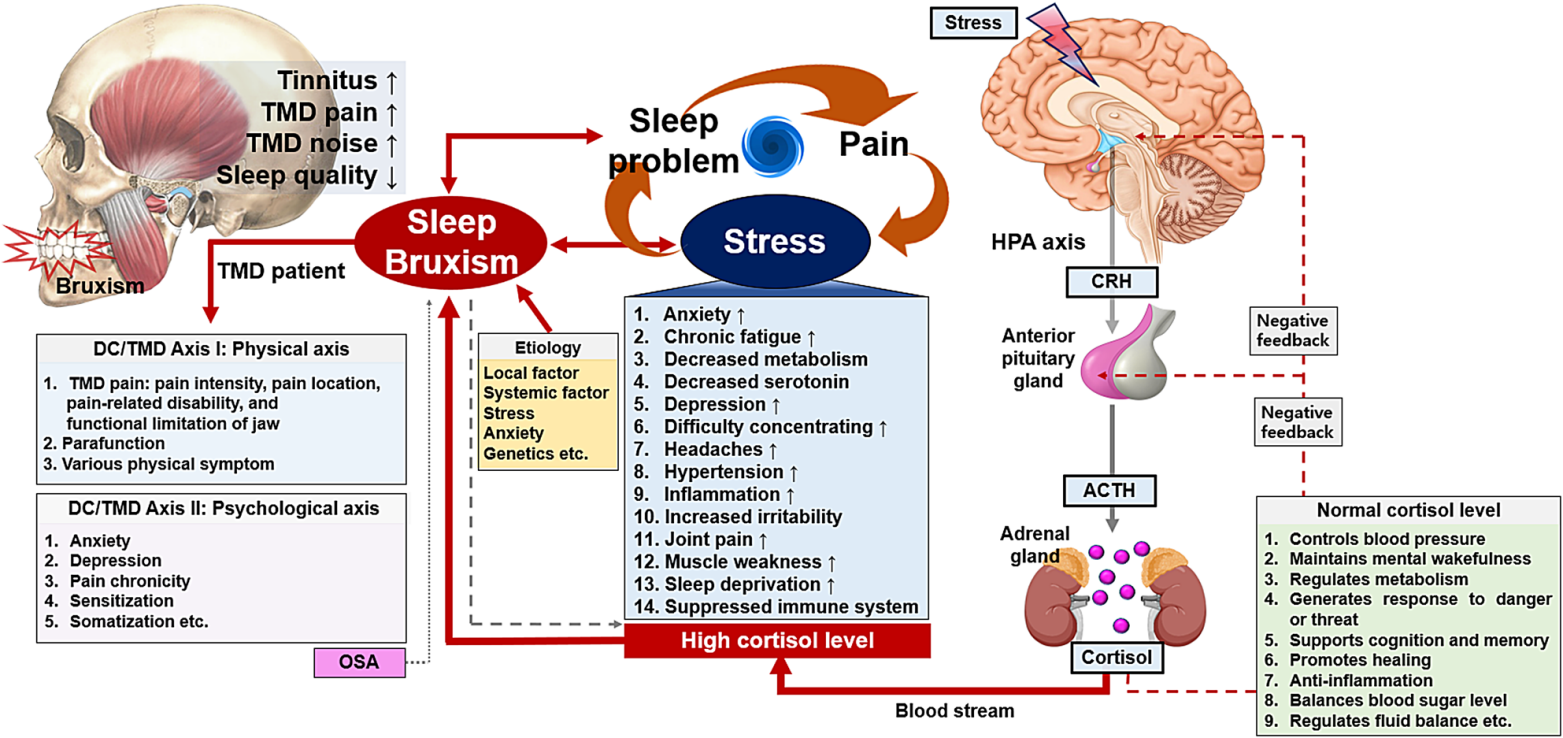
Diagram of the relationships among sleep bruxism, TMD pain, psychological stress, and cortisol levels.* TMD* temporomandibular disorder,* DC/TMD* diagnostic criteria for temporomandibular disorders,* OSA* obstructive sleep apnea,* HPA axis* hypothalamic–pituitary–adrenal axis,* CRH* corticotropin-releasing hormone,* ACTH* adrenocorticotropic hormone.

In this study, we investigated the clinical, sleep-related, psychological, and hematological factors associated with SB in patients with TMD, identified significant predictors of SB, and explored the complex interrelationships among these variables (Fig. 4). In line with our expectations, the results supported our initial hypotheses. Patients with TMD and SB exhibited more severe clinical symptoms, poorer sleep quality, greater psychological distress, and elevated levels of cortisol and ACTH. Furthermore, an inverse association between age and the presence of SB was observed, consistent with our hypothesis that younger patients would be more likely to exhibit SB. These findings underscore the importance of comprehensive, multidimensional assessment in patients with TMD, particularly in those suspected of having SB. Early identification of psychological distress, poor sleep quality, and hormonal dysregulation may aid in developing targeted and effective management strategies.

SB was correlated with several clinical factors, including TMJ noise, TMD pain, pain intensity (CMI), LMO, and tinnitus. SB was also associated with elevated CAR, ACTH, and cortisol levels. Psychological distress has also been linked to SB. Further analysis revealed complex interconnections between SB and its predictors. For predicting SB in patients with TMD, the factors arranged in descending order of AUC values are as follows: factors with acceptable predictive performance—CMI, ACTH, and DEP; factors with relatively weak predictive performance—ANX, PSQI global score, cortisol, and CAR. Conversely, the cutoff value for age was 50 years, indicating a higher likelihood of the absence of bruxism in individuals aged 50 years or older. Factors associated with an increased likelihood of SB were the pain intensity, poor sleep quality, DEP, ANX, CAR ratio, and ACTH levels. Notably, an increase in age was associated with a decreased likelihood of SB, with a cut-off value of 50 years, indicating a significant decrease in bruxism occurrence in individuals aged ≥ 50 years. Bruxism can occur at all ages; however, its prevalence peaks between 45 and 54 years^53^.

The significance of the co-occurrence of SB and TMD requires verification using objective scientific data. SB is a prevalent condition, with an estimated occurrence of 31–70% in the general population^15,54^ Among them, one in four individuals with associated symptoms are aware of the condition^55^ In this study, the incidence of SB in patients with TMD was 46.83%. According to the OPPERA study, TMD pain and oral parafunctions, including bruxism, are potential risk factors for chronic TMD^56^ Complications associated with SB include TMD, tooth wear, tooth sensitivity, periodontal tissue damage, masticatory muscle fatigue and soreness, and facial and ear pain^1,57^Multiple logistic regression analysis identified pain intensity as the strongest predictor of an increased likelihood of SB (OR = 18.400), followed by poor sleep quality (OR = 11.425), DEP (OR = 1.189), cortisol/ACTH ratio (CAR) (OR = 1.151), ANX (OR = 1.081), and ACTH (OR = 1.073). Therefore, it is difficult to conclude that TMD pain is solely caused by SB. Moreover, CMI demonstrated notable predictive accuracy for SB in patients with TMD, indicating that pain intensity is a significant predictor of SB in this population. According to Fernandes et al., SB may be a risk factor for painful TMD^58^; however, the occurrence of TMD pain is not associated with the intensity of SB^59^ Additionally, noncontinuous bruxism may help alleviate physical tension and psychological stress and provide positive stimulation to the brain^60,61^ These findings reflect the complex and bidirectional nature of the relationship between SB and TMD. Establishing a cause-effect relationship among these factors remains challenging, highlighting the need for additional research in this area.

Beyond its local musculoskeletal manifestations, SB may also exert systemic effects through neuroendocrine pathways and stress-related physiological responses. This perspective extends our understanding of SB beyond the orofacial region, framing it as a condition with potential multisystem implications. SB, especially when involving repetitive mechanical loading of the masticatory muscles and subsequent activation of the HPA axis, may be associated with systemic inflammation and oxidative stress^54,62^. These physical and biochemical stressors, as components of the broader stress-related cascade, are known to contribute to the pathogenesis of various chronic conditions, including autoimmune diseases, hypertension, and cardiovascular disorders^63^. Cortisol and ACTH–both examined in this study–are well-recognized mediators of the neuroendocrine stress response, which may, in turn, promote inflammatory and oxidative processes. A recent polysomnography-based study further supports this association, demonstrating elevated markers of oxidative stress in individuals with SB^64^. Although the present study did not directly assess inflammatory or oxidative biomarkers, the observed neuroendocrine alterations in SB patients with TMD may reflect broader systemic physiological changes. With these mechanisms, SB may contribute not only to the persistence and chronicity of TMD symptoms but also to the complex interplay between TMD and systemic inflammatory conditions. Such possibilities warrant further investigation in future studies exploring the biological consequences of SB.

Oral parafunction, and hormonal and psychosocial factors can aggravate TMD pain^65^ During bruxism, persistent and repeated physical movements of the mandible can result in soreness, fatigue, and pain in the TMJ and masticatory muscles^66^Additionally, the repetitive motion of the mandible can damage the TMJ structure, eventually leading to TMJ noise and pain. Furthermore, owing to the close anatomical proximity of the internal auditory canal and TMJ^67^, mechanical stimulation of the TMJ from bruxism and physiological stimulation from inflammation may be implicated in the pathogenesis of tinnitus or otalgia. Conversely, TMD symptoms, such as pain, TMJ noise, and tinnitus may strongly indicate the presence of SB. However, in a recent systematic review, osteoarthritic pain was found to be weakly correlated with elevated cortisol levels^68^ Therefore, understanding patients with TMD requires understanding not only the physical factors of axis I but also the psychological aspects responsible for axis II and the neuroendocrine response products of the stress-related HPA axis, such as cortisol and ACTH.

Cortisol, a quintessential stress hormone, is synthesized and secreted by the HPA axis in response to stress and other noxious stimuli. Upon stimulation, the hypothalamus secretes CRH, which promotes the release of ACTH from the pituitary gland. ACTH stimulates cortisol secretion from the adrenal cortex. Facial pain may serve as a more potent stimulus for HPA axis activation than pain originating in other regions of the body^69^ In patients with TMD, elevated cortisol level is a significant predictor of SB and is correlated with psychological stress and sleep problems. Higher-than-average levels of ACTH and CAR increased the odds of SB occurrence in patients with TMD by 1.073 and 1.151 times, respectively. Predicting SB in patients with TMD using ACTH levels yielded acceptable predictive accuracy (AUC = 0.708, cut-off value 28.575 pg/mL), whereas using cortisol levels resulted in acceptable predictive accuracy (AUC = 0.643, cut-off value 7.105 µg/dL). Corticosterone- or stress-dependent behavioral changes are accompanied by neurochemical and neuroanatomic alterations^70^However, given the variability in individual HPA axis responses to stress^71^, plasma cortisol or ACTH should be used carefully as biomarkers for stress or bruxism in patients with TMD. In patients with susceptible psychological profiles or chronic TMD, the HPA axis can influence the pathophysiological progression of the disorder^72^ In this study, cortisol levels were significantly higher in patients with TMD and bruxism than in those without bruxism. Additionally, SB was associated with depression, anxiety, sleep disorders, and psychological stress. Patients with TMD and depression had higher salivary cortisol levels than those without depression^73^ However, there is insufficient research to clearly elucidate and delineate the complex relationships between these factors.

Elevated cortisol levels may indicate the presence of pain or sustained psychological stress and may be correlated with associated human behaviors. The relationship between pain (both acute and chronic), HPA axis function, and its subsequent effect on cortisol levels has been extensively documented^74,75^ Pain acts as a potential stressor and activator of the HPA axis. Depending on the perceived threat level associated with pain, physiological responses may be exaggerated, resulting in cortisol dysfunction^76^ Chronic joint inflammation can perpetuate a stress response, leading to cortisol dysfunction and widespread inflammation, which subsequently contribute to chronic disability^68^ Neuroendocrine associations between pain and hormonal responses mediated by the HPA axis are frequently linked to psychological distress. Psychological stress and sleep are intricately linked, and increased stress and sleep loss interact to induce hyperactivation of the HPA axis. Sleep deprivation and sleep disorders are associated with maladaptive alterations in the HPA axis, resulting in neuroendocrine dysregulation. The salivary cortisol levels in patients with TMD were higher than those of the control group^77^ However, studies on these factors in patients with TMD are limited. Thus, cortisol secretion, psychological stress, pain, and sleep deterioration mediated by the HPA axis in patients with TMD should be comprehensively investigated.

Previous studies have shown that stress and poor sleep quality are important contributing factors not only to bruxism but also to the development and exacerbation of TMD^78-81^. Stress-related activation of the masticatory muscles and dysregulation of the HPA axis may contribute to both TMD pain and SB^78^. Moreover, sleep disturbances are known to interfere with pain modulation, thereby aggravating musculoskeletal symptoms associated with TMD^12^. SB is also associated with poor sleep quality in patients with TMD. Specifically, patients with TMD with bruxism had higher PSQI scores than those without bruxism, with a cut-off score of 10.5 predicting the presence of bruxism. Poor sleep quality, high DEP scores, and traumatic childhood experiences increase the risk of bruxism^82,83^ Sleep is crucial for survival, body function, and pain management. Adequate sleep is essential for maintaining one’s overall health and well-being. SB can disrupt the macrostructure of sleep, leading to adverse changes in the total sleep time, sleep latency, and sleep efficiency. Consequently, it impairs sleep quality and architecture^84^ Awake bruxism may indicate nervousness, anxiety, or depression, which can adversely affect sleep quality and duration. This reduction in sleep quantity and quality, coupled with psychological issues, can create a cycle that exacerbates symptoms in patients with TMD and bruxism. However, SB does not cause these serious problems in all individuals, and some posit that bruxism itself is not a pathology but rather a mechanism for stress relief^85^ Therefore, when treating patients with TMD and bruxism, it is imperative to thoroughly evaluate their sleep status and neuroendocrine responses.

Although OSA has been posited as an emerging risk factor for SB^86^, the data on the association between SB and OSA remain inconclusive and contradictory. In this study, a high risk of OSA was not correlated with the presence of SB in patients with TMD. SB, psychological problems, and even sleep disturbances may share underlying mechanisms, such as disruption of the brain’s gamma-aminobutyric acid (GABA) and glutamatergic systems^87,88^Although GABA, an inhibitory neurotransmitter, is essential for inducing and maintaining sleep, SB is associated with attenuated GABAergic activity^87^, which may lead to increased microarousal and cardiac sympathetic activity. Low GABA activity leads to anxiety, depression, and insomnia, which can be resolved using GABAergic drugs^89^ HPA dysfunction can play a significant role in the manifestation of bruxism, stress, and anxiety in individuals with TMD^54^ These alterations in the neuroendocrine system may further complicate the pain profiles of patients with TMD.

A limitation of this study is the relatively small sample size in relation to the wide range of parameters examined. Although statistical analysis was feasible, a larger multicenter follow-up study is required to support our findings. The extended study duration, which exceeded four years, was primarily due to the challenges of administering multiple questionnaires and blood tests to patients with TMD in a clinical setting, as well as the difficulty of obtaining complete datasets suitable for analysis. We measured cortisol levels in blood samples from patients with TMD to examine their associations with SB and other factors. Although we attempted to control for potential confounding variables―such as medication use, menstrual cycle status, and individual stress levels―not all factors could be systematically assessed or incorporated into the analysis. Notably, our sample included a disproportionately higher number of female participants. Considering that sex hormones such as estrogen and progesterone may influence pain perception and stress-related pathways^90,91^, and given our findings on ACTH and cortisol, future research should explore the interplay between neuroendocrine responses, hormonal status, and SB in TMD populations. Moreover, selection bias may have occurred due to the invasive nature of hematological testing. The diagnosis of SB in this study was based on self-reported symptoms and clinical indicators, rather than confirmation through polysomnography, which remains the gold standard as recommended by the American Academy of Sleep Medicine^92^. According to the STAB criteria^41^, this approach corresponds to possible SB rather than a definitive diagnosis. While the STAB framework is rigorous, it may be considered overly strict for typical observational settings. Readers are therefore advised to interpret the results conservatively, avoiding overgeneralization beyond the classification of possible SB. Nonetheless, a recent study suggests that portable polysomnography may serve as a feasible and less resource-intensive alternative for SB screening in clinical research settings^93^. Both TMD and SB have complex and multifactorial etiologies, many aspects of which remain poorly understood. Despite these limitations, further investigation into the diverse contributing factors of SB in patients with TMD remains essential.

Various factors that correlate with SB in patients with TMD remain insufficiently explored. Hence, the elucidation of the mechanisms underlying our primary findings and systematic scientific exposition of the complex interrelationships among the above factors necessitates further research.

## Conclusion

The findings of this study substantiate the idea that clinical factors, including patient age, TMD pain, sleep deterioration, psychological distress, and endocrine responses, as indicated by cortisol and ACTH levels, are associated with SB and may serve as potential indicators in patients with TMD. Specifically, SB in TMD patients is linked to age < 50 years, TMD pain, TMJ noise, poor sleep quality, psychological distress, and elevated cortisol and ACTH levels. Complex interconnections are also observed among these factors. Sleep-related factors are significantly associated with an increase in the PSQI global score and the presence of poor sleepers. SB is further linked to psychological distress. Increases in cortisol, ACTH, and CAR levels have also been linked to SB. Cortisol showed only weak predictive ability for SB, whereas ACTH demonstrated an acceptable level of discrimination. Nonetheless, both cortisol and ACTH, as markers of endocrine response, are intrinsically associated with SB.

## Data Availability

The datasets used and/or analyzed in the current study are available from the corresponding author upon reasonable request.
